# Changes in the Human Gut Microbiota Associated With Colonization by *Blastocystis* sp. and *Entamoeba* spp. in Non-Industrialized Populations

**DOI:** 10.3389/fcimb.2021.533528

**Published:** 2021-03-18

**Authors:** Gaël Even, Ana Lokmer, Jules Rodrigues, Christophe Audebert, Eric Viscogliosi, Laure Ségurel, Magali Chabé

**Affiliations:** ^1^ Gènes Diffusion, Douai, France; ^2^ PEGASE-Biosciences, Institut Pasteur de Lille, Lille, France; ^3^ UMR7206 Eco-Anthropologie, CNRS-MNHN-Université de Paris, Paris, France; ^4^ Univ. Lille, CNRS, Inserm, CHU Lille, Institut Pasteur de Lille, U1019-UMR 9017-CIIL-Centre d’Infection et d’Immunité de Lille, Lille, France; ^5^ Laboratoire de Biométrie et Biologie Evolutive UMR5558, CNRS - Université Lyon 1, Université de Lyon, Villeurbanne, France

**Keywords:** *Blastocystis*, *Entamoeba*, gut microbiota, 16S rRNA gene, metagenomics, Cameroon, intestinal protozoa

## Abstract

Human gut microbial communities are mainly composed of bacteria, but also include fungi, viruses, archaea, and protozoa, whose role in the gut ecosystem has only recently begun to be recognized. For example, humans colonized by *Blastocystis* (a gut protozoan with controversial pathogenicity) host a more diverse bacterial microbiota than individuals not carrying it, suggesting that its presence may be beneficial for the host. In parallel, the presence of non-pathogenic *Entamoeba* spp. has been associated with an increased diversity and compositional shifts in the bacterial microbiota of healthy rural individuals in Cameroon. However, *Entamoeba* and *Blastocystis*, the two most prevalent human gut protozoa, have never been studied in the same individuals, preventing the study of their interaction. As *Blastocystis* is one of the few gut protozoa commonly found in industrialized populations, which are otherwise mostly devoid of gut eukaryotes, we need to focus on rural “traditional” populations, who harbor a higher diversity of gut eukaryotes (whether pathogenic or commensal) in order to study protozoa interactions in the gut ecosystem. To this end, we profiled the gut bacterial microbiota of 134 healthy Cameroonian adults using 16S rRNA gene amplicon sequencing data. *Entamoeba* and *Blastocystis* presence and co-occurrence pattern in the same individuals were determined using metagenomic shotgun data. We found that, when taking into account both protozoa jointly, *Blastocystis* was associated with both a higher richness and a higher evenness of the gut bacterial microbiota, while *Entamoeba* was associated only with a higher richness. We demonstrated a cumulative influence of these protozoa on bacterial microbiome diversity. Furthermore, while the abundance of several common taxa (for example, *Ruminococcaceae*, *Coprococcus* and *Butyrivibrio*) varied according to *Blastocystis* colonization, only a single *Bacteroides* amplicon sequence variant was found to be differentially abundant between *Entamoeba*-negative and *Entamoeba*-positive samples. Given the specific signature of each protozoan on the gut microbiota and the seemingly stronger association for *Blastocystis*, our results suggest that *Blastocystis* and *Entamoeba* interact with gut bacteria each in its own way, but experimental studies are needed to explore the precise mechanisms of these interactions.

## Introduction

Trans-kingdom interactions have undoubtedly shaped human gut homeostasis due to millions of years of coevolution (Jackson et al., 2009). For example, some bacterial and eukaryotic microorganisms residing in the human gut can affect each other’s pathogenicity (Leung et al., 2018). However, most studies on eukaryote-microbiota interactions so far have focused on well-known pathogenic protozoa such as *Giardia*, *Cryptosporidium*, and *Entamoeba histolytica* (Burgess et al., 2017; Leung et al., 2018), the only exception being (Chudnovskiy et al., 2016), who demonstrated the protective effect of a non-pathogenic protozoan *Tritrichomonas musculis* against *Salmonella typhimurium* enterocolitis in mice (Chudnovskiy et al., 2016). In addition, little is known about the ecological interactions between gut bacteria and intestinal protozoa that are either non-pathogenic or whose pathogenicity is unknown or controversial, and who might even be beneficial (Scanlan et al., 2014; Lukeš et al., 2015; Chabé et al., 2017).

These potentially beneficial gut eukaryotes include *Blastocystis* and *Entamoeba* (excluding the pathogenic *E. histolytica*), whose prevalence in humans exceeds that of other protozoa (Lokmer et al., 2019). *Blastocystis* is a cosmopolitan unicellular eukaryote historically considered as a parasite (Stensvold and Clark, 2016), but whose pathogenicity is nowadays disputed (Scanlan and Stensvold, 2013; Lukeš et al., 2015; Chabé et al., 2017; Stensvold and van der Giezen, 2018). The studies carried out mainly in industrialized countries reported a higher diversity of gut bacterial microbiome in colonized individuals, regardless of the subtype (ST) (Audebert et al., 2016; Tito et al., 2019). In addition, *Blastocystis* colonization seems to be associated with a higher relative abundance of intestinal bacteria that are usually indicative of a healthy gut microbiota (Andersen and Stensvold, 2016; Audebert et al., 2016; Iebba et al., 2016; Beghini et al., 2017; Nieves-Ramírez et al., 2018; Kodio et al., 2019). Regarding *Entamoeba* spp. other than pathogenic *E. histolytica*, their effect on health is even less clear, but some arguments can be made for their beneficial effect (Morton et al., 2015; Iebba et al., 2016). Indeed, in the only study so far considering the influence of non-pathogenic *Entamoeba* spp. (later shown to be mostly *E. coli, E. dispar*, and *E. hartmanni* by Lokmer et al, 2019) on the gut microbiota, Morton et al. (2015) observed a higher bacterial microbiota diversity in individuals colonized by non-pathogenic *Entamoeba*, as well as a lower relative abundance of bacteria usually correlated with inflammatory or autoimmune diseases. Furthermore, Iebba et al. (2016) found that individuals from Côte d’Ivoire carrying *E. coli*, *E. hartmanni*, and/or *E. dispar* had a high *Faecalibacterium prausnitzii*-*Escherichia coli* ratio, usually associated with eubiosis.

Interestingly, in the only study to this date examining both *Blastocystis* and *Entamoeba* in the same subjects, these two protozoa were observed together more often than by chance (Lokmer et al., 2019). It is thus possible that the changes in gut bacterial diversity and composition observed in *Entamoeba*-positive individuals are partly due to the colonization by *Blastocystis*, or vice-versa. To disentangle the relationship between bacterial microbiota and each of the two protozoa, we need to study their influence in the same human cohort. Furthermore, the studies published so far have shown shifts in composition associated with each protozoan separately, but it is not clear if the same bacterial genera are implicated in these compositional shifts (as differences in methods between studies make their comparison non-trivial). Comparing their association with gut microbiota composition in the same cohort might thus help us to better understand the underlying processes. Indeed, if both protozoa influence the host immune system, which then indirectly influences the gut bacteria, they might be associated with similar compositional shifts. Another hypothesis is that the presence of *Blastocystis* and non-pathogenic *Entamoeba* simply reflects a gut microbiota that is prompt to “receive” them, in which case the two protozoa would simply represent healthy gut indicators (Stensvold and van der Giezen, 2018). However, if each protozoan directly influences the gut bacteria in its own way (whether by predation (Mosca et al., 2016) or by secretion of compounds), we might expect that their effects on diversity and composition would differ.

It therefore seems relevant to study the potential interaction of these two intestinal protozoa in the same population in order to determine whether their presence has a comparable impact on the gut microbiota from colonized individuals. To achieve this, given that *Entamoeba* colonization is rare (if not absent) in industrialized countries, our study focused on the intestinal microbiota of healthy adults from rural, semi-urban and urban areas in Cameroon. In this study, we used the 16S rRNA gene amplicon sequencing data from 134 Cameroonians generated in a previous survey (Lokmer et al., 2020) to profile the bacterial composition and diversity of their gut microbiota. The detection and identification of *Blastocystis* STs and *Entamoeba* species was performed by analyzing shotgun metagenomic data from the same individuals generated for this study.

## Methods

### Sampling and Ethics Statement

Fecal samples were collected from 134 healthy adults (59 males and 75 females), living in three different areas of Cameroon (76 in rural, 26 in semi-urban and 32 in urban environments). The research permits, including the appropriate ethic approvals, were obtained for this study from the CNERSH (Comité National d’Ethique de la Recherche pour la Santé Humaine) in Cameroon (Approval n°2017/05/900), as well as from regional health districts (Centre region, Approval n°0061). We further obtained an ethical approval from the French CPP (Comité de Protection des Personnes, approval n°2016-sept-14344), as well as the authorization to import and store these samples from the French Ministry of Higher Education and Research (n°IE-2016-876 and DC-2016-2740, respectively). Finally, we obtained the authorization to store personal data in France from the CNIL (Commission Nationale Informatique et Libertés, n°1972648). We obtained the informed consent of each participant for contributing to this research. These samples are part of the dataset previously published (Lokmer et al., 2020). The average age was 38 years with a range between 18 and 64 years. Body mass index (BMI) was calculated for all the individuals who were categorized as underweight, normal weight, overweight or obese if they had a BMI < 18.5 (n = 9), from 18.5 to 24.9 (*n* = 89), ≥ 25 (n = 26) or ≥ 30 (n = 9), respectively. BMI information was not available for one subject and age information was missing for another one (**Supplementary Table 1**). Age and sex distribution as well as demographic data according to the colonization by *Blastocystis* STs and *Entamoeba* species are given in **Supplementary File 1**.

### DNA Extraction and Sequencing

Total DNA was extracted from approximately 250 mg of each fecal sample (homogenized by bead beating) using the MOBIO PowerFecal DNA Isolation Kit (MOBIO Laboratories, Carlsbad, CA, USA) according to the manufacturer’s protocol. DNA isolated from fecal samples was quantified using a NanoDrop (ThermoScienctific). Sequencing libraries targeting the V4 region of the 16S rRNA gene were prepared by the Microbial Omics Core (MOC) facility at the Broad Institute, Cambridge, MA, USA and sequenced as 150bp paired-end reads in two runs on an Illumina MiSeq 300 (as described in (Poyet et al., 2019), resulting in an average of 50,802 ± 25,076 (st. dev.) raw reads per sample. Shotgun sequencing libraries were prepared using Nextera XT DNA Library Prep Kit (Illumina, San Diego, CA, USA) and sequenced as 125 bp paired-end reads on a HiSeq2000 sequencer (Illumina) at the University of Minnesota Genomics Center (Minneapolis, MN, USA). We obtained an average of 23.6 million read pairs per sample. Raw data have been deposited in European Nucleotide Archive under the study accession numbers PRJEB30836 (16S rRNA gene amplicon data) and PRJEB30834 (Whole Genome Sequencing data, WGS).

### Sequencing Data Quality Control and Preprocessing

To process raw amplicon reads, QIIME2 (Bolyen et al., 2019) microbiome analysis package has been used. Full documentation of all analysis code in this project can be found at https://rachaellappan.github.io/VL-QIIME2- analysis/. Briefly, raw fastq files were first converted into QIIME2 compatible files. After a quality check, DADA2 (Callahan et al., 2016) was used for calling amplicon sequence variant (ASVs). DADA2 uses a statistical error correction model and attempts to remove or correct reads with sequencing errors and then remove chimeric sequences originating from different DNA templates. We used SILVA database v132 (Quast et al., 2013) as the reference database in combination with QIIME2’s pre-fitted sklearn-based taxonomy classifier tool for ASV taxonomic classification. *De novo* phylogenetic trees used to calculate phylogenetic diversity were also built using default QIIME2 provided methods, i.e., align-to-tree-mafft-fastree (MAFFT multiple sequence alignment program) (Price et al., 2010; Katoh and Standley, 2013). A total of 2768 ASVs were obtained after pre-processing.

WGS data were preprocessed as described in (Lokmer et al., 2019). Briefly, we filtered the reads from low-quality areas of the flowcell, trimmed the adapters and low quality bases (Phred Q<10) using BBMap package (Bushnell, 2017), leading to 42 ± 5.5 million reads per sample.

### Statistical Analyses

Alpha (within-sample) diversity was measured using observed ASVs, Pielou’s index, Shannon’s H and Faith’s Phylogenetic Diversity (PD) calculated in QIIME2 from rarefied counts. Although the rarefaction curve (**Supplementary Figure 1**) of Shannon’s H reached a plateau after 2,000 reads, we rarified the data to 10,000 in order to provide robustness in diversity analyses. This resulted in loss of 20 samples with read depths <10,000 reads, leaving a sample size of 114 for downstream analyses.

In order to visualize the relationship between gut microbiome diversity, colonization by protozoa and contextual variables, we performed a FAMD (Factor Analysis of Mixed Data) including quantitative (Age, BMI, Faith’s PD index) and qualitative variables (*Blastocystis* and *Entamoeba* colonization status, sex and lifestyle). FAMD was calculated using R (R Development Core Team and Team, 2005) and the package *FactoMineR* (Lê et al., 2008).

Boxplots showing alpha diversity were created in QIIME2. To test for the conditional effect of each protozoan as well as the interactions of both protozoa on alpha diversity, we fitted generalized least squares (gls) linear models (~*Blastocystis*+*Entamoeba* + *Blastocystis : Entamoeba*), after testing for heteroscedasticity and specifying the variance structure in case it was significantly different, using the *nlme* package in R (Pinheiro et al., 2019). To assess beta (between-sample) diversity, Bray-Curtis dissimilarities, weighted and unweighted UniFrac distances were calculated on rarefied counts. Principal coordinates analysis (PCoA) 2D plots for these indices were generated in QIIME2 using the EMPeror graphics tools (Vázquez-Baeza et al., 2013). The effect of protozoan colonization on the beta diversity has been examined by PERMANOVA implemented in the R package *vegan* (Oksanen et al., 2013), number of permutations = 999). We used ANCOM in QIIME2 to determine differences in ASV abundances between four groups of individuals (i.e., B-/E-, B+/E-, B-/E+, and B+/E+) or between B+/B- and E+/E-. Relative differential abundance test was conducted in STAMP v 2.0.9 (Parks et al., 2014) at taxonomic levels from species to phylum, using two-sided Welch’s t-test for pairwise comparisons. The taxa have been considered as differentially abundant if the Benjamini–Hochberg-corrected p-value (i.e., q-value) ≤ 0.05. Co-occurence analysis was performed using the R package *coocurr* (Griffith et al., 2016).

### Detection of *Blastocystis* and *Entamoeba* by Metagenomic Data

The presence of *Blastocystis* and *Entamoeba* was assessed by mapping metagenomic shotgun data to respective genomes or 18S rRNA gene sequences using BWA-MEM (Li, 2013) as described in (Lokmer et al., 2019). Briefly, for *Blastocystis*, we used the whole genomes of all relevant subtypes (ST1-ST9). We only considered read pairs where both reads mapped to the same genome. We filtered the alignments based on the amount of soft clipping, edit distance and alignment length as described in (Lokmer et al., 2019).

For *Entamoeba*, we used the available 18S rRNA gene sequences for all relevant species (*E. histolytica*, *E. dispar, E. hartmanni, E. coli, E. moshkovskii*), as there are no publicly available genomes for the majority of *Entamoeba* species. Contrarily to the approach for whole genomes, to define whether an individual was positive or negative for *Entamoeba* colonization, we kept all reads that mapped, even if they mapped to multiple *Entamoeba* species or if only one read mate of a given read pair mapped (**Supplementary Table 2**). While most reads mapped to a single species, and thus most individuals could be unambiguously assigned to the species level, we detected one individual for which the species could not be determined (labelled as E. undetermined) (**Supplementary Table 2**). Regarding *E. histolytica*, all reads that mapped to its 18S rRNA also mapped to the 18S rRNA sequence of *E. dispar* (reflecting their high sequence similarity), but the reverse was not true (results not shown). To confirm the absence of *E. histolytica*, we mapped all reads to its whole genome and found no positive individuals according to our filtering criteria. Thus, we concluded that *E. histolytica* was never found in these individuals (as was also the case in the cohort studied in (Lokmer et al., 2019). Thus, we removed it from subsequent analyses (i.e., we remapped the reads to 18S rRNA gene sequences of non-pathogenic species only).

## Results

### Prevalence of Blastocystis and Entamoeba

A total of 134 fecal samples from healthy Cameroonian adults were screened for *Blastocystis* sp. and *Entamoeba* spp. by mapping metagenomic shotgun reads (23.6 million read pairs in average per sample) on the protozoan sequences (see Methods). 72 of these individuals (53.7%) were positive for both protozoa (**Supplementary Table 1**).

*Blastocystis* was found in 75.4% of the 134 subjects while *Entamoeba* was detected in 59.7% of these individuals. *Blastocystis* ST3 was the most prevalent (42.5%), followed by ST1 (33.6%) and ST2 (23.9%). ST4 was also observed, but at much lower prevalence (0.7%) (**Supplementary Table 1**). In parallel, *E. coli* and *E. hartmanni* were the most prevalent *Entamoeba* species (35.1% and 34.3%, respectively), then followed by *E. dispar* (16.4%) (**Supplementary Table 1**). We did not find any individual positive for *E. histolytica* (see Methods).

**Figure 1:**
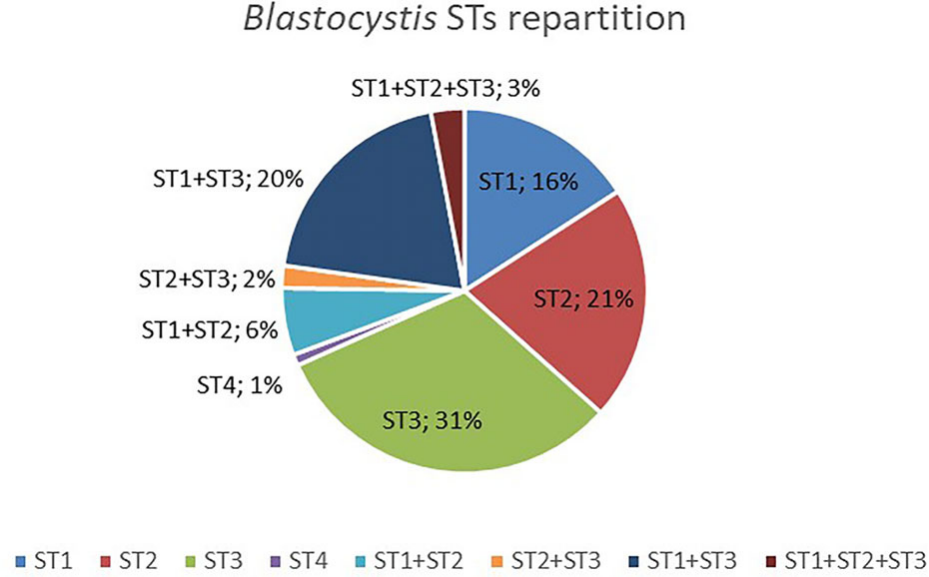
Distribution of *Blastocystis* STs detected in colonized Cameroonian individuals.

Among those colonized by *Blastocystis*, most individuals (69%) were colonized by only one ST (**Figure 1**), but 31% had mixed infections (with two or three different STs). ST1-ST3 was the most prevalent combination (20%) (**Figure 1**). Interestingly, mixed infections with three STs (i.e., ST1, ST2 and ST3) were all found in rural individuals. No significant associations have been found between the presence of *Blastocystis* or its STs colonization (i.e., when classified by ST1, ST2, ST3, or mixed infection) and BMI, age, or sex (Kruskal-Wallis test and Fisher’s exact test, p > 0.05). However, we found a trend toward a lower prevalence in urban individuals (62.5%) as compared to rural and semi-urban individuals (77.6 and 84.6%, respectively) (chi-square test, p = 0.105 and p = 0.061, respectively).

**Figure 2:**
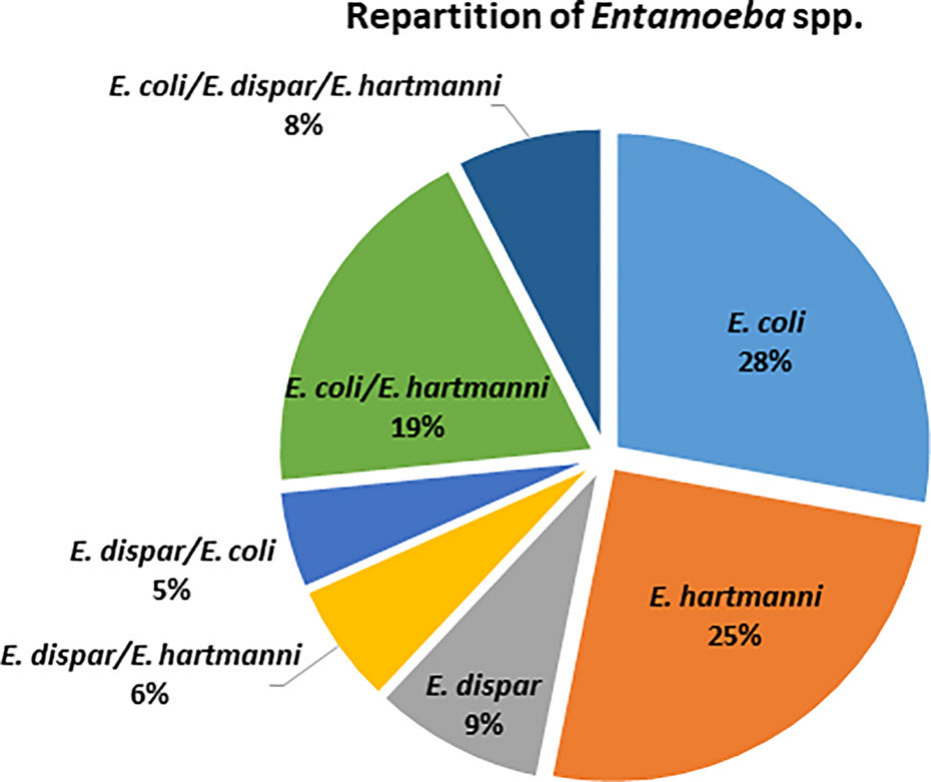
Distribution of identified *Entamoeba* species detected in colonized Cameroonian individuals.

Among the 79 individuals colonized by an identified species of *Entamoeba*, 38% were colonized by at least two species of *Entamoeba* (**Figure 2**), with the majority being colonized by *E. coli*/*E. hartmanni* (19%) or all three of them (8%). Among single infections, *E. coli* and *E. hartmanii* were the most prevalent (28% and 25%, respectively). *Entamoeba* colonization was not found to be significantly associated with either BMI or age (Kruskal-Wallis test, p > 0.05). However, women were found to be more likely than men to be colonized by *Entamoeba* (Fisher’s exact test, p = 0.034). Moreover, *Entamoeba* prevalence seemed to correlate with the urbanization level, with a higher percentage of colonized individual in rural (71.1%) than in semi-urban (50%) and urban (40.6%) areas (chi-square test, p = 0.153 and p = 0.003, respectively).

### Association Patterns Between Protozoan Species

Overall, 72 out of 134 individuals (53.7%) were positive for both *Blastocystis* and *Entamoeba*. A chi-square test of independence showed that there was a significant positive relationship between *Blastocystis*- and *Entamoeba*-colonization status (p < 0.001), indicating that the individuals carrying one of the two protozoa were more likely to be colonized by the other. Although the presence of *Blastocystis* and *Entamoeba* were significantly correlated, the strength of association was low (Cramer’s V = 0.001) and they were thus considered as two independent variables in the linear models.

**Figure 3:**
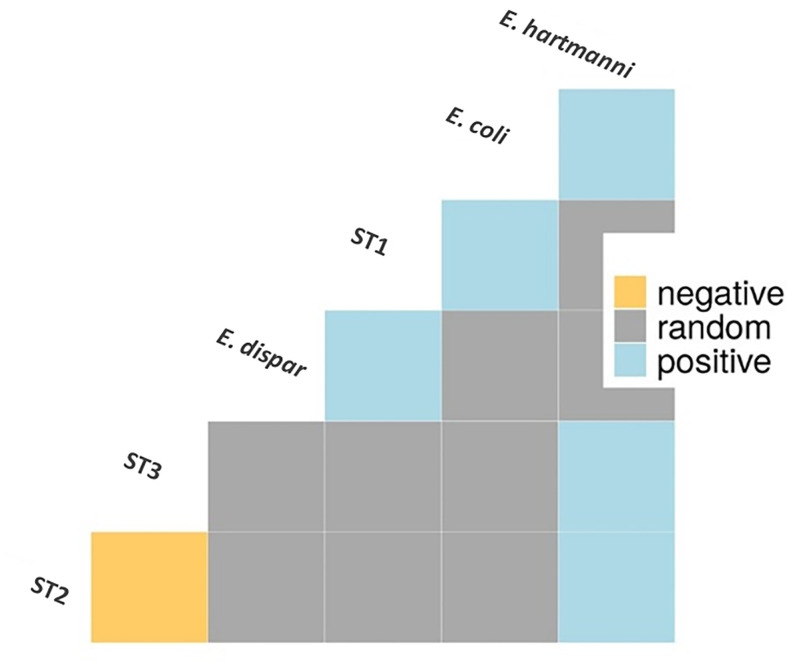
Association patterns between *Entamoeba* species and *Blastocystis* subtypes in Cameroonian individuals. Only species/subtypes with at least one significant correlation are shown. E, *Entamoeba*; ST, subtype.

A co-occurrence analysis was performed to examine association patterns between *Blastocystis* STs 1-4 and *E. coli*, *E. hartmanni* and *E. dispar*. Out of 21 possible pairwise combinations, six significant associations have been detected (five positives and one negative) involving six protozoan species/STs (**Figure 3**). Specifically, *Blastocystis* ST1 was positively correlated with *E. coli* and *E. dispar*; *Blastocystis* ST2 and ST3 were both positively associated with *E. hartmanni*. In addition, we identified a positive association between two *Entamoeba* species, *E. coli* and *E. hartmanni*. Conversely, *Blastocystis* ST2 and ST3 appeared negatively correlated (**Figure 3**).

### Relationship Between Protozoan Colonization and Alpha Diversity of the Gut Microbiome

To evaluate different aspects of alpha diversity, four diversity indices have been calculated from the 16S data (mean 50,802 raw reads per sample): observed ASVs, Faith’s Phylogenetic Diversity (Faith’s PD), Shannon’s H, and Pielou’s evenness. A FAMD (Factor Analysis of Mixed Data) was first used to identify which contextual variables were positively or negatively associated with the bacterial microbiome diversity. Overall, age, BMI, sex and urbanization level were not correlated with Faith’s PD (**Supplementary Figure 2**) or any other diversity indices (results not shown) in our study.

**Figure 4:**
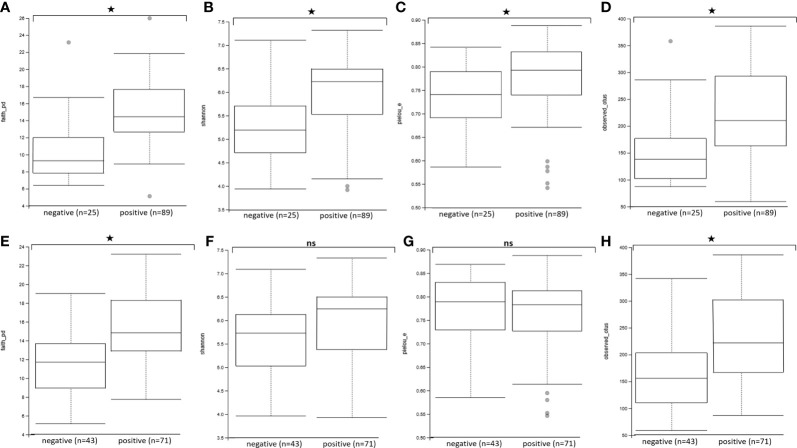
Comparison of alpha diversity indices between *Blastocystis*-positive and *Blastocystis*-free groups **(A–D)** and *Entamoeba*-positive and *Entamoeba*-negative groups **(E–H)**. **(A, E)** Faith’s Phylogenetic Diversity (faith_pd). **(B, F)** Shannon index (shannon). **(C, G)** Pielou’s evenness index (pielou_e). **(D, H)** observed ASVs (observed_asvs). ns, not significant. *p < 0.05.

**Figure 5:**
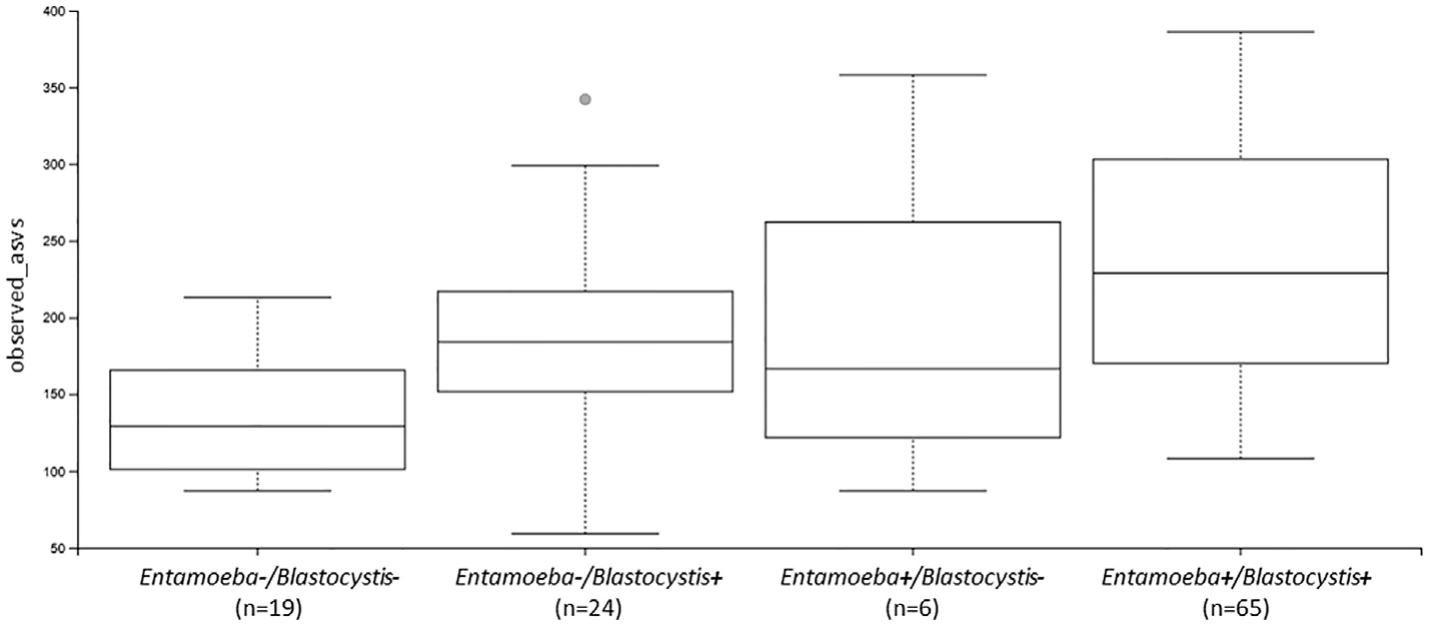
Boxplots showing the differences in the number of observed ASVs between individuals colonized by no protozoan, only *Blastocystis*, only *Entamoeba* or both protozoa (Ent, *Entamoeba*; Blasto, *Blastocystis*).

We then assessed the effect of *Blastocystis* and *Entamoeba* jointly (using *gls* linear models taking into account both variables and their interaction) on the alpha diversity of the gut microbiota (**Figure 4**, **Figure 5**, **Supplementary Figure 3**). All four calculated diversity indices were significantly higher in the presence of *Blastocystis* (gls ANOVA: p < 0.01 for each index, **Supplementary Table 3**), with a 3% and 6% increase in evenness and Shannon’s H, respectively, and a 11% and 13% increase in Faith’s PD and the number of observed ASVs, respectively (parameter estimates with CI for *Blastocystis*_colonization are in **Supplementary Table 3**). Similarly, the presence of *Entamoeba* was associated with a 13% and 15% increase in Faith’s PD and observed ASVs, respectively (gls ANOVA: p < 0.01, parameter estimates for *Entamoeba* colonization, **Supplementary Table 3**). However, no significant difference has been found between the *Entamoeba-*free and *Entamoeba-*colonized groups for Shannon and Pielou’s evenness (gls ANOVA, p = 0.273 and p = 0.557, respectively, **Supplementary Table 3**). In addition, the fitted linear models provided no evidence of significant *Blastocystis* x *Entamoeba* interaction for any of the diversity indices, indicating that the presence of *Blastocystis* and *Entamoeba* affect the gut microbiome diversity in a cumulative way, independently of each other (**Supplementary Table 3**).

**Figure 6:**
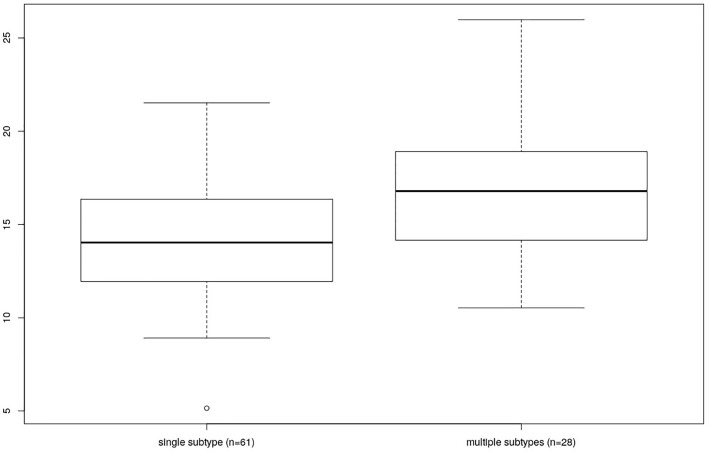
Comparison of Faith’s PD between individuals differing by the number of colonizing *Blastocystis* subtypes (single vs. multiple).

Finally, the individuals colonized by multiple *Blastocytis* STs were characterized by a higher bacterial alpha diversity compared to the individuals colonized by a single *Blastocystis* ST, whether or not they were also colonized by *Entamoeba* (Kruskal-Wallis test, p = 0.006, see **Figure 6** and **Supplementary Figure 4** for Faith’s PD; results were similar for other alpha diversity indices, data not shown). No significant difference was found between the bacterial alpha diversity from individuals colonized by multiple *Entamoeba* species (N=58) compared to the individuals colonized by a single *Entamoeba* species (N=13) (Kruskal-Wallis test, p = 0.202, for Faith’s PD). We chose not to assess the effect of individual *Blastocystis* ST or specific *Entamoeba* species on diversity given that we do not have enough power (i.e., individuals) to do so.

### Relationship Between Protozoan Colonization and the Gut Microbiome Beta Diversity

**Figure 7:**
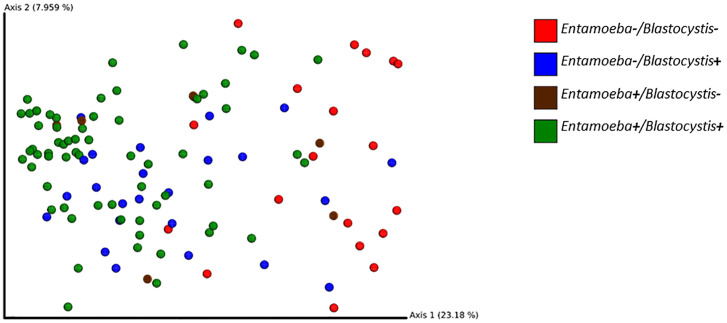
2D EMPeror beta diversity plot snapshot representing the first two principal components of the PCoA analysis (unweighted Unifrac matrix).

To investigate the effect of protozoa colonization on the variability of the gut bacterial microbiome, we performed PCoA on Bray-Curtis dissimilarities and unweighted or weighted Unifrac distances. These PCoA showed a modest clustering of the samples based on their *Blastocystis*-or *Entamoeba*-colonization status regardless of the metric used (**Figure 7**). However, a PERMANOVA analysis showed a significant difference between the *Blastocystis-*positive and *Blastocystis*-negative communities for both Bray-Curtis, weighted and unweighted Unifrac distances, albeit the amount of variability explained was low (R2 = 1.6%, p = 0.010; R2 = 2.5%, p = 0.046 and R2 = 3.6%, p = 0.001, respectively, **Supplementary Table 4**). Similarly, 1.4% of the variability in Bray-Curtis and 2.8% of that in unweighted Unifrac distances was explained by *Entamoeba* colonization (p = 0.016 and p = 0.001, respectively, **Supplementary Table 4**), whereas weighted Unifrac distances did not differ between the *Entamoeba-*positive and *Entamoeba*-negative individuals (p = 0.304). Furthermore, beta diversity was not affected by the interaction between the two protozoa regardless of the dissimilarity measure (p = 0.074, p = 0.208 and p = 0.079, respectively).

### Relationship Between Protozoan Colonization and the Gut Bacterial Microbiome Composition

To investigate the effect of *Blastocystis* and *Entamoeba* presence on the gut microbiome composition in more detail, we performed an ANCOM analysis at the ASV level, as well as STAMP analyses on taxonomic levels ranging from species to phylum. Specifically, we were interested in differences between four colonization groups, i.e., B-/E-, B+/E-, B-/E+, and B+/E, and in the differences between B+/B- and E+/E-. All the mean relative abundances of bacterial classes, orders, families and genera in each group of individuals are listed in **Supplementary Table 5**.

According to the ANCOM analysis, the relative abundance of two ASVs from the *Ruminococcaceae* UCG-002 genus differed between the four colonization groups, being less abundant in the protozoa-free group (**Supplementary Table 6**). When examining the effect of *Blastocystis* and *Entamoeba* colonization separately, we found the same two ASVs (as well as another one belonging to the genus *Ruminococcaceae* UCG-005) to be significantly higher in individuals carrying *Blastocystis*. In addition, we found a lower abundance of a *Bacteroides* ASV in *Entamoeba*-positive individuals (**Supplementary Table 6**).

STAMP analyses revealed no difference between *Entamoeba*-positive and *Entamoeba*-negative samples (Welch’s t-test, q > 0.05). Concerning *Blastocystis*, a higher proportion of the orders Mollicutes RF39, Methanobacteriales, Elusimicrobiales and Izimaplasmatales has been detected in the subjects colonized by *Blastocystis* (q < 0.05). At the family level, *Ruminococcaceae* had almost double relative abundance in the colonized (18.2%) than in the *Blastocystis*-negative group (10.2%, q < 0.05, **Table 1**). Similarly, different *Ruminococcaceae* were more abundant in the *Blastocystis-*positive individuals at the genus level (**Table 1**). Other genera that were more abundant in the *Blastocystis*-positive group (q < 0.05) include: *Butyrivibrio*, *Christensenellaceae* R-7 group, *Elusimicrobium*, *Coprococcus*, *Eubacterium ruminantium*, and *Eubacterium xylanophilum* groups (**Table 1**).

**Table 1:** Mean relative abundances of various bacterial families and genera in *Blastocystis*-positive and *Blastocystis*-negative individuals.

	*Blastocystis* colonization status		
	Positive (n = 89)	Negative (n = 25)	q value (FDR adjusted p value)
*Ruminococcaceae*	18.2%	10.2%	q = 8.74e-3
*Ruminococcus^a^*	11.6%	4.9%	NA^b^
*Coprococcus* 2	2.3%	0.8%	q = 6.44e-3
*Butyrivibrio*	1.3%	0.1%	q = 1.04e-3
*Elusimicrobium*	0.56%	0.04%	q = 7.02e-3
*Christensenellaceae* R-7 group	0.55%	0.13%	q = 5.46e-3
*Eubacterium ruminantium* group	0.84%	0.24%	q = 0.015
*Eubacterium xylanophilum* group	3.4e-04%	3.5e-05%	q = 9.60e-3

NA, not applicable.

^a^Corresponds to the addition of mean relative abundances of D_5:Ruminococcaceae NK4A214 group, D_5:Ruminococcaceae UCG-002, D_5:Ruminococcaceae UCG-003, D_5:Ruminococcaceae UCG-005, D_5:Ruminococcaceae UCG-008, D_5:Ruminococcaceae UCG-009, D_5:Ruminococcaceae UCG-010, D_5:Ruminococcaceae UCG-013, D_5:Ruminococcaceae UCG-014, D_5:Ruminococcus 1 and D_5:Ruminococcus 2 ASVs.

^b^Significant differences between the two groups were found for D_5:Ruminococcaceae UCG-002, D_5:Ruminococcaceae UCG-005, D_5:Ruminococcaceae UCG-010, D_5:Ruminococcaceae UCG-014, D_5:Ruminococcus 1 and D_5:Ruminococcus 2 ASVs.

Welsh t-tests with Benjamini-Hochberg with False Discovery Rate multiple test correction generated q values (FDR adjusted p values) in STAMP.

## Discussion

There is an increasing evidence that intestinal non-pathogenic protozoa can affect the bacterial gut microbiota. Indeed, several studies on *Blastocystis* (Andersen et al., 2015; Audebert et al., 2016; Nieves-Ramírez et al., 2018; Kodio et al., 2019) and two on non-pathogenic *Entamoeba* (Morton et al., 2015; Lokmer et al., 2019) demonstrated the association of these two protozoa with changes in the gut bacterial microbiome diversity and composition. However, no study so far examined the influence of their simultaneous presence in the gut. To fill this gap, we investigated the effect of *Blastocystis* and *Entamoeba* colonization on the intestinal microbiota of Cameroonian individuals, a population with a high reported prevalence of these two protozoa (Lokmer et al., 2019).

First, we confirmed that *Blastocystis* colonization has a significant impact on the diversity and composition of the gut bacterial microbiota in a population with a low level of industrialization. The presence of *Blastocystis* was indeed associated with a higher diversity of the gut bacterial microbiota, regardless of the alpha-diversity index studied (richness or evenness). This result is consistent with the studies carried out on *Blastocystis* so far (Andersen et al., 2015; Audebert et al., 2016; Forsell et al., 2017; Nash et al., 2017; Kodio et al., 2019), with the exception of (Beghini et al., 2017), who found no change in Shannon and Gini-Simpson indices depending on *Blastocystis* colonization on in any of the examined populations. Similarly, the structure of the bacterial gut communities differed between *Blastocystis*-colonized and *Blastocystis*-free groups here, regardless of the beta-diversity index used. Although our study was not designed (and thus underpowered) to test for potential differences in bacterial microbiome composition depending on the *Blastocystis* ST, we have been able to show, for the first time, that the diversity of the gut bacterial microbiota is higher in subjects colonized by several *Blastocystis* STs than in individuals carrying only one ST. Whereas this correlation between *Blastocystis* diversity (i.e., the number of STs an individual is colonized by) and the richness of the bacterial microbiome could be explained by individual differences in exposure to microbes due to personal hygiene habits or sanitary conditions, this would not explain the higher bacterial evenness found in people with multiple *Blastocystis* STs. Explanation of this phenomenon thus requires further investigation in cohorts with larger sample sizes.

Regarding the specific compositional shifts, several bacterial taxa were significantly enriched in *Blastocystis*-positive individuals. We found a higher relative abundance of *Ruminococcaceae*, similar to a number of previous studies (Andersen et al., 2015; Beghini et al., 2017; Kodio et al., 2019). This is also in line with the observation that *Blastocystis* carriage is less prevalent in individuals with a *Bacteroides*-driven enterotype than in those with a *Ruminococcus*- or *Prevotella*-driven enterotype (Stensvold and van der Giezen, 2018; Tito et al., 2019). We also observed a higher (albeit statistically non-significant) relative abundance of the order *Clostridiales* in *Blastocystis*-positive individuals (44.1% vs. 34.0% in *Blastocystis*-free ones, q = 0.105), consistent with a study from France (Audebert et al., 2016) and with studies performed in less industrialized countries (Beghini et al., 2017; Kodio et al., 2019). All the other bacterial taxa that were enriched in *Blastocystis*-colonized individuals in the Cameroonian population were found by (Beghini et al., 2017) and (Kodio et al., 2019) as well, highlighting a good consistency across studies. Finally, it is interesting to note that many of these taxa (i.e., *Ruminococcaceae*, *Coprococcus*, *Butyrivibrio*, and *Christensenellaceae*) include butyrate-producing bacteria, known to play a key role in gut health of humans (Louis and Flint, 2009). In line with this finding, we found no microbial signature of dysbiosis (like a bloom of Proteobacteria (Winter and Bäumler, 2014) in *Blastocystis*-positive individuals.

Therefore, if we consider that the presence of *Blastocystis* (i) is rarely associated with symptoms in colonized individuals; (ii) is associated with the increased relative abundance of likely beneficial bacteria and with a higher microbiome diversity, considered a hallmark of a healthy gut (Le Chatelier et al., 2013) as well as (iii) with the absence of intestinal inflammation (Nieves-Ramírez et al., 2018), it seems that colonization by *Blastocystis* is not deleterious to the human intestinal microbiota, and may even be beneficial. Accordingly, the exclusion of stool from *Blastocystis* positive donors with the aim of performing fecal microbiota transplantation (FMT) has recently been questioned by (Terveer et al., 2019). They found that transferring feces containing *Blastocystis* ST1 and ST3 did not result in gastrointestinal symptoms in recipients and did not affect the outcome of FMT treatment.

On the other hand, we found that the effect of *Entamoeba* on the gut bacterial microbiota is different, and less pronounced, than that of *Blastocystis*. Indeed, whereas we found an increase in species and lineage richness (observed ASVs and Faith’s PD) in *Entamoeba*-positive individuals, we did not find significant differences in evenness estimations (Pielou or Shannon’s H). Accordingly, while *Entamoeba* presence significantly affected both the Bray-Curtis dissimilarities and unweighted Unifrac distances (though modestly), it did not affect weighted Unifrac distances. Finally, *Entamoeba* colonization was only associated with the significant change of a single ASV (a decrease in *Bacteroides*). We thus argue that *Entamoeba* interact with the gut microbiota through a different mechanism than *Blastocystis*. Notably, it seems to introduce only fine-scale (ASV level) changes and primarily influence rare bacteria.

In the introduction, we hypothesized that if the changes in the diversity and composition of bacterial microbiota differed between the two protozoa (which is the case), it could reflect differences in bacteria-protozoa interactions between these intestinal eukaryotes. This could, for example, suggest that the interaction is mediated through bacterial predation. Unfortunately, almost nothing is known about the identity and quantities of bacteria ingested by either *Blastocystis* or non-pathogenic *Entamoeba* (Dunn et al., 1989; Hamad et al., 2017). This highlights the need to investigate direct interactions between these non-pathogenic protozoa and the bacteria in the gut in order to better understand how they shape gut microbial communities.

We can also hypothesize that *Blastocystis* and *Entamoeba* thrive in different niches, implying distinct interactions with other inhabitants of the gut microbiota that are known to be non-uniformly distributed along the gastrointestinal tract (Tropini et al., 2017). *E. histolytica* trophozoites colonize the large intestine, especially the cecal and sigmoidorectal regions. For *Blastocystis*, studies in rodent models and naturally infected pigs have shown that the protozoan localizes to the lumen and mucosal surface of the large intestine mostly in the cecum and colon. Occasionally, in immunosuppressed pigs, *Blastocystis* can also be detected in the small intestine (Ajjampur and Tan, 2016).

While we can strongly suggest that *Blastocystis* and *Entamoeba* likely act through different mechanisms to interact with the gut bacterial microbiota, our study is observational and thus focused on correlations and cannot unveil the causal relationships between gut protozoa and bacterial microbiota. In order to uncover mechanisms by which protozoa colonization influences the intestinal microbiota, we will need laboratory experiments, using, for example, new in-*vitro* models such as gut-on-a-chip (Jalili-Firoozinezhad et al., 2019; Poceviciute and Ismagilov, 2019) or intestine 3-D models combined with omics and other techniques such as microscopy (Aguilar-Rojas et al., 2020). Direct interactions of protozoa with gut bacteria can also be studied by cultivation experiments using synthetic gut microbiomes (Vrancken et al., 2019) and protozoa of interest. In addition, longitudinal studies before and after protozoan colonization in animal models could provide important insights into protozoa-bacterial microbiome interactions in a more natural but still highly controlled settings (Leung et al., 2018). Finally, our work demonstrates the need to account for the presence of intestinal eukaryotes such as *Blastocystis* when studying the interaction of *Entamoeba* spp. amoebas with the intestinal bacterial microbiota.

## Data Availability Statement

The datasets generated for this study can be found in NCBI PRJEB30834: accessions: ERS052637-ERS3052903.

## Ethics Statement

The studies involving human participants were reviewed and approved by the research permits, including the appropriate ethic approvals, and were obtained for this study from the CNERSH (Comité National d’Ethique de la Recherche pour la Santé Humaine) in Cameroon (Approval n°2017/05/900), as well as from regional health districts (Centre region, Approval n°0061). We further obtained an ethical approval from the French CPP (Comité de Protection des Personnes, approval n°2016-sept-14344), as well as the authorization to import and store these samples from the French Ministry of Higher Education and Research (n°IE-2016-876 and DC-2016-2740, respectively). Finally, we obtained the authorization to store personal data in France from the CNIL (Commission Nationale Informatique et Libertés, n°1972648). We obtained the informed consent of each participant for contributing to this research. The patients/participants provided their written informed consent to participate in this study.

## Author Contributions

AL, GE, EV, MC, and LS contributed to the conception of the work. AL, JR, GE, and CA performed the data curation. GE, AL, CA, MC, and LS designed the methodology. Formal analyses were done by GE and AL. MC, AL, and LS wrote the paper and all the authors revised it. All authors contributed to the article and approved the submitted version.

## Funding

This work was supported by the ANR (Agence Nationale de la Recherche) grant MICROREGAL (ANR-15-CE02-0003), as well as by a research price IDF-FRM (Institut Danone France—Fondation pour la Recherche Médicale) attributed to LS.

## Conflict of Interest

GE and CA are employed by the company Gènes Diffusion.

The remaining authors declare that the research was conducted in the absence of any commercial or financial relationships that could be construed as a potential conflict of interest.
