# Supplementary material for: Changes in the Human Gut Microbiota Associated With Colonization by Blastocystis sp. and Entamoeba spp. in Non-Industrialized Populations
Source: Front Cell Infect Microbiol. 2021 Mar 18;11:533528. doi: 10.3389/fcimb.2021.533528 (PMC8013780; doi:10.3389/fcimb.2021.533528)
Supplement: Supplementary file 1 [file DataSheet_1.docx]

Supplementary Material

**Supplementary File 1.**

Distribution of Cameroonian samples by age group and sex:

|  | 18-29 | 30-39 | 40-49 | 50-64 | Total |
| --- | --- | --- | --- | --- | --- |
| Male No. (%) | 16 (27.59%) | 12 (20.69%) | 15 (25.86%) | 15 (25.86%) | 58 (43.61%) |
| Female No. (%) | 21 (28.00%) | 20 (26.67%) | 21 (28.00%) | 13 (17.33%) | 75 (56.39%) |
| Total | 37 (27.82%) | 32 (24.06%) | 36 (27.07%) | 28 (21.05%) | 133 (100%) |

Age information was not available for one individual from the study.

Descriptive statistics of Cameroonian individuals grouped by *Blastocystis* colonization status and subtype (ST):

|  | *Blastocystis* colonized | ST1 only | ST2 only | ST3 only | Mixed infection |
| --- | --- | --- | --- | --- | --- |
|  | (n = 101) | (n = 16) | (n = 21) | (n = 32) | (n = 31) |
| Female % | 56.4% | 56.25% | 66.7% | 42.9% | 58.1% |
| Age: mean (SD) | 38.34 (10.99) | 35.19 (8.64) | 37.33 (12.30) | 38.97 (10.89) | 39.52 (11.25) |
| BMI: mean (SD) | 23.06 (4.19) | 22.22 (3.69) | 23.05 (4.42) | 23.51 (4.43) | 23.25 (4.06) |

SD: standard deviation. The only individual colonized by *Blastocystis* ST4 is not mentioned in this table.

Descriptive statistics of Cameroonian individuals grouped by *Entamoeba* colonization status and species:

|  | *Entamoeba* colonized | *E. coli* only | *E. hartmanii* only | *E.* *dispar* only | Mixed infection |
| --- | --- | --- | --- | --- | --- |
|  | (n = 79) | (n = 22) | (n = 20) | (n = 7) | (n = 30) |
| Female % | 63.3% | 54.5% | 70% | 28.6% | 73.3% |
| Age: mean (SD) | 38.94 (10.64) | 43.32 (10.41) | 37.8 (10.13) | 38.43 (8.40) | 36.60 (10.64) |
| BMI: mean (SD) | 23.20 (4.25) | 22.48 (3.73) | 24.39 (4.69) | 23.26 (1.55) | 22.89 (4.53) |

SD: standard deviation. We did not consider the only individual colonized by an undetermined *Entamoeba* species according to our analysis pipeline.

**Supplementary Figure 1**. Rarefaction curves for Shannon’s diversity indices in the Cameroonian population according to their *Blastocystis* (a) and *Entamoeba* (b) colonization status.

a)


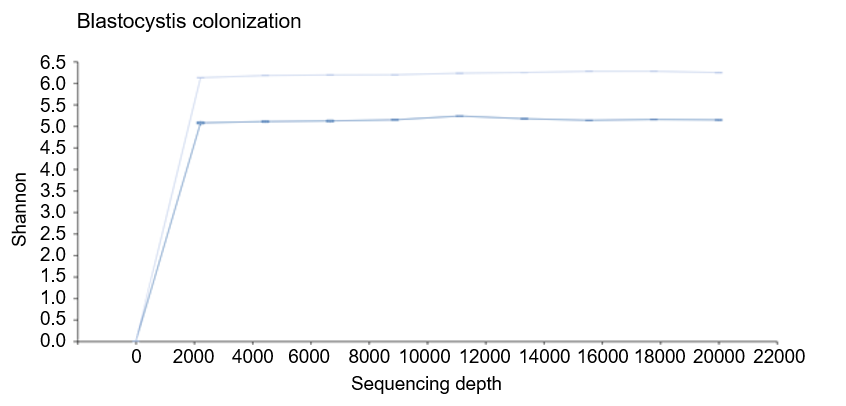


b)


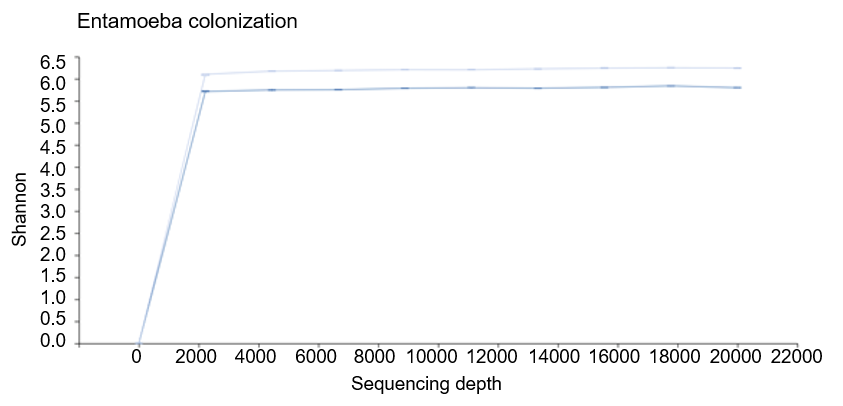


The light blue lines represent *Blastocystis*- and *Entamoeba*-positive samples and the dark blue lines represent the *Blastocystis*- and *Entamoeba*-free samples.

**Supplementary Figure 2**. Correlation circle graph from the Factor Analysis of Mixed Data (FAMD) including quantitative (age, Faith’s PD index, BMI) and qualitative (*Blastocystis* and *Entamoeba* colonization status, sex and lifestyle) variables from the Cameroonian individuals.


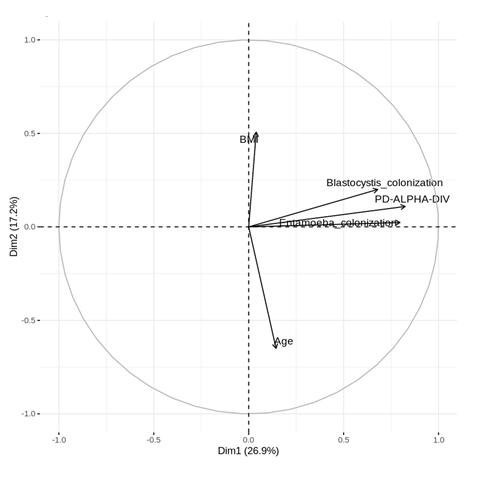


BMI = Body Mass Index; PD-ALPHA-DIV = Faith’s PD (Phylogenetic Distance) index.

Sex and lifestyle were taken into account in the FAMD analysis but are not graphically represented here.

**Supplementary Figure 3**. Boxplots comparing Shannon’s (A), Pielou’s (B) and Faith’s PD (C) indexes in *Blastocystis*- and/or *Entamoeba*-colonized groups and protozoan-free group.

(A)


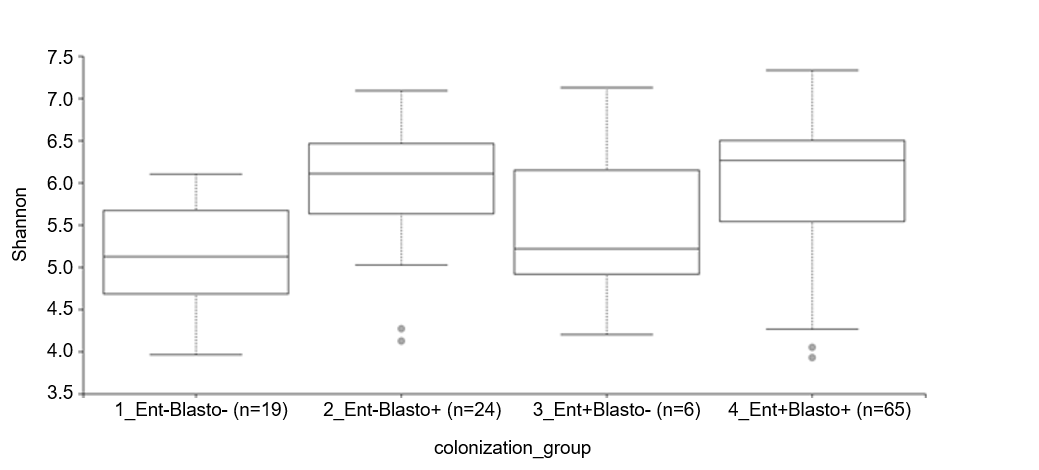


(B)


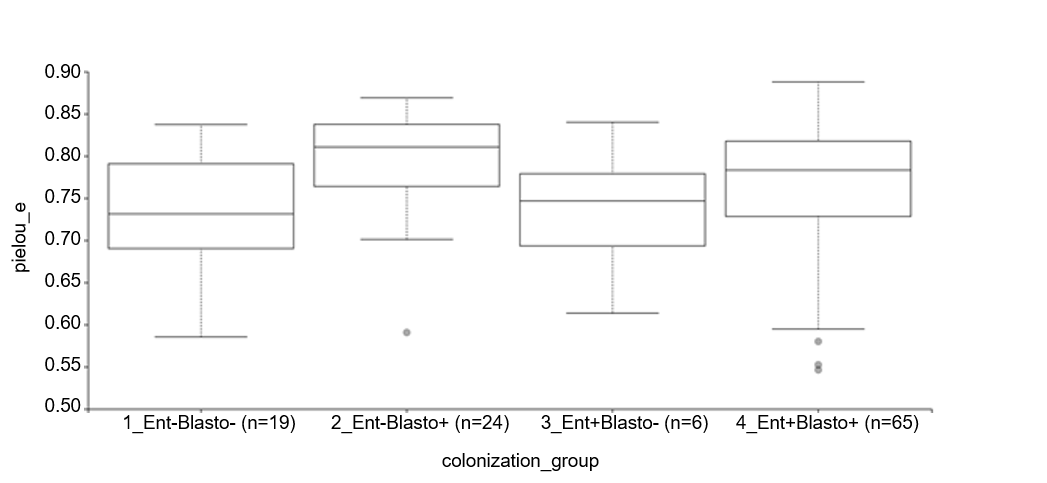


(C)


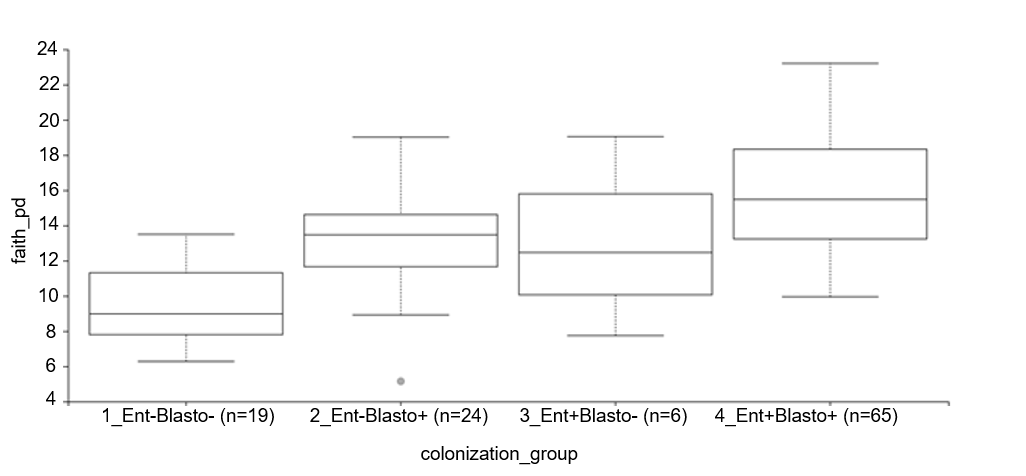


**Supplementary Figure 4**. Comparison of mean Faith’s PD diversity measures for *Entamoeba*-free individuals colonized by multiple *Blastocystis* subtypes or by a single subtype (p = 0.0365, Kruskal-Wallis test).


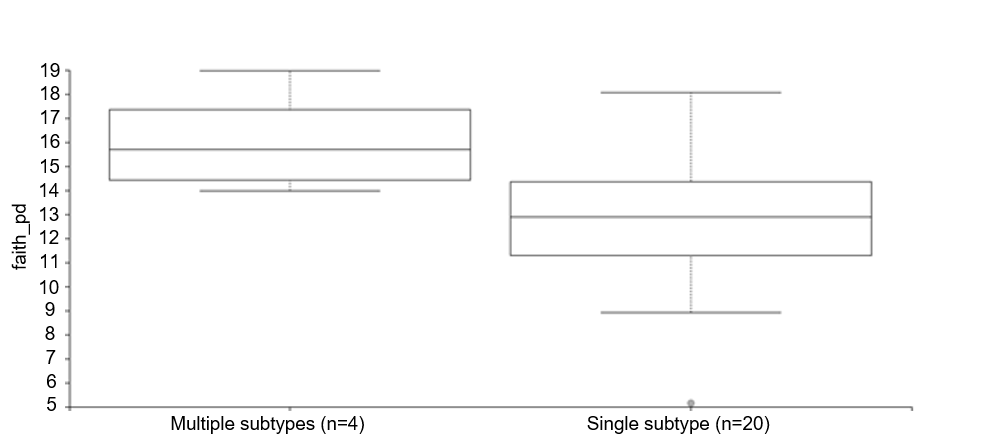


**Supplementary Table 1**: Metadata from the Cameroonian individuals included in this study. Information about *Blastocystis* STs and *Entamoeba* spp. colonization status, sex, age, BMI, alpha-diversity indices’ means and number of filtered reads is given.

**Supplementary Table 2:** Number of hits, positive contigs, proportion of positive contigs and breadth of coverage for *Blastocystis* and *E. histolytica* genomes and number of filtered 18S rRNA gene hits for other *Entamoeba* species. In both cases, the raw alignments were filtered for almost perfect hits. The reads mapping to more than one genome were removed. For genomes, only the samples with breadth of coverage >=0.001 and proportion of positive contigs >=0.1 were considered positive. For *Entamoeba*, samples with any hits were considered positive (due to very strict prefiltering). For more details, see Lokmer *et al*. 2019 Plos One.

**Supplementary Table 3:**gls ANOVA results for the four alpha-diversity indices (observed OTUs, Shannon’s, Pielou’s and Faith’s PD).

**Supplementary Table 4:** PERMANOVA results for the three beta-diversity measures (Bray-Curtis, unweighted and weighted Unifrac distances) and *Blastocystis* x *Entamoeba* interactions.

**Supplementary Table 5:**mean relative abundances of bacterial taxa from the gut microbiota (levels 3, 4, 5 and 6 corresponding respectively to Class, Order, Family and Genus) according to *Blastocystis* and *Entamoeba* colonization.

**Supplementary Table 6:** Results from ANCOM analysis of between-group differences in microbial composition. The w Ancom metric denotes the number of sub-hypotheses H0 that are rejected during the multiple pairwise comparison tests used by ANCOM,
